# The Role of Estrogen in Brain MicroRNAs Regulation

**DOI:** 10.34172/apb.39216

**Published:** 2024-09-15

**Authors:** Peyvand Bahramiazar, Naseh Abdollahzade, Bakhtyar Tartibian, Naser Ahmadiasl, Fakhreddin Yaghoob Nezhad

**Affiliations:** ^1^Department of Physiology, Tabriz University of Medical Sciences, Tabriz, Iran.; ^2^Neurophysiology Research Center, Cellular and Molecular Medicine Institute, Urmia University of Medical Sciences, Urmia, Iran.; ^3^Department of Exercise Physiology, Faculty of Physical Education and Sport Sciences, Allameh Tabataba’i University, Tehran, Iran.; ^4^TUM School of Medicine and Health, Technical University of Munich, Germany.

**Keywords:** miRNA, Estrogen, Brain, Aging, Ovariectomy

## Abstract

**Purpose:**

This review aims to elucidate the role of estrogen-sensitive microRNAs (miRNAs) in modulating brain functions and disorders, highlighting the protective effects of estrogen on the central nervous system.

**Methods:**

A comprehensive literature review was conducted, examining the relationship between estrogen, miRNAs, and cognitive health. The study focused on experimental data comparing cognitive impairments between genders and the mechanisms of estrogen’s effects on brain function.

**Results:**

Cognitive impairments are less prevalent in women of reproductive age compared to men, indicating estrogen’s neuroprotective role. Estrogen modulates gene expression through specific receptors, while miRNAs regulate approximately 30% of protein-coding genes in mammals. These miRNAs play critical roles in synaptic plasticity and neuronal survival. The review identifies several estrogen-sensitive miRNAs and their potential involvement in brain disorders.

**Conclusion:**

The interplay between estrogen and miRNAs offers valuable insights into the molecular mechanisms underlying cognitive health and disease. Understanding these relationships may lead to novel therapeutic strategies for addressing various brain disorders, particularly those associated with hormonal changes and aging.

## Introduction

 Brain and neurological disorders are among the major causes of mortality and disability throughout the world. According to recent investigations, the incidence of these diseases will increase in the next decades.^1^ The incidence of diabetes, psychiatric problems, hypertension, and degenerative brain diseases, such as dementia and stroke, is more frequently seen in elderly people. Lack of effective treatment leads to turning these diseases into a major problem.^2^ Although it is not clear how age contributes to these diseases, studies have shown cognitive complaints are common in women, who are transitioning through menopause.^3^ It is important, therefore, to understand the effects of normal ovarian aging and menopause on the CNS. Both experimental and clinical data have revealed the benefits of estrogen in the brain.^4^ Estrogen has neurotrophic and neuroprotective properties and is necessary for conserving learning and memory.^5^ It has also been shown estradiol attenuates reactive oxygen species release,^6^ inhibits the production of macrophage cytokines, decreases expression of the inducible isoform of nitric oxide synthases, and increases anti-inflammatory signaling pathways.^7^ Various studies have indicated that ovariectomy (OVX) increases brain damage and neurodegenerative processes as a result of decreasing estrogen levels.^8^ Furthermore, hormonal therapy restores the estradiol levels and recovers brain damage in ovariectomized animals.^9^ It is proven that estrogen controls various signaling pathways through its receptors,^10^ and subsequently, regulates the expression of microRNAs (miRNAs) in most tissues such as the brain.^11^ miRNAs regulate protein translation by binding their target messenger RNAs (mRNAs) and inhibiting the translating or degrading mRNA molecules.^12^ Emerging evidence shows that miRNAs have nuclear functions at the transcriptional level in terms of regulating gene expression.^13^ Thus, miRNA-mediated regulation is now considered one of the most important post-transcriptional gene regulation mechanisms and regulates more than 30% of mammal genes, including important roles in human physiology, aging, and CNS disorders.^14^ Even though many studies have suggested miRNAs as crucial epigenetic regulators of brain function, their role in regulating estrogen in brain disorders is not fully understood. This review aims to identify current knowledge of the role of estrogens in brain function with a special focus on the miRNAs that are regulated by estrogens.

## MiRNA functions and processing overview

 MiRNAs are a class of small (~20-22 nucleotide) non-coding RNAs that appear to have been extensively researched from the time of their discovery by Ambros,^15^ and Ruvkun,^16^ in *Caenorhabditis elegans* and Baulcombe,^17^ in plants and their prominent role in cardiovascular disease, diabetes, cancers and aging disorders has been shown.

 Recent evidence indicates that miRNAs play important roles in regulating various cellular processes including translation of select mRNAs, apoptosis, differentiation, and replication.^18-20^ The human genome contains around 2588 mature miRNAs.^21^ About 50% of miRNAs are intragenic and most of them are found within the 50 introns of host genes.^22^ In studies, it is estimated that 1-4% of the human genome is made up of miRNAs, and one miRNA can regulate about 200 mRNAs,^23^ and since they are involved in many human biological processes, their regulation may be disturbed. lead to widespread disorders in the body, so that about 70 miRNA-related diseases have been reported (http://cmbi.bjmu.edu.cn/hmdd). It is likely that the reports of these cases will increase over time. The miRBase reference repository currently contains information on 1917 human precursors and 2656 mature miRNAs (version 22).^24^ that this figure is increasing day by day, although a number of false positive cases have also been reported among them, which the reference databases try to minimize these cases, so that there is a significant reduction of miRNAs with high confidence in the new versions of miRBase Is. However, the quality of relevant miRNA databases ultimately always depends on the availability of highly reliable positive and negative training sets, i.e., miRNAs validated by appropriate experimental methods. and requires global definition of criteria.^25,26^ However, based on experiments listed in miRBase, there is evidence of miRNA expression by NB for only 3.6% of all human miRNAs listed in miRBase V22.^27-29^ And recently, the studies of Alles et al,^30^ who used an experimental method with high efficiency and reliability and in different tissues, data showed 2300 mature human miRNAs, 50% of them were also included in miRBase V22. Although this number cannot be final, based on the data presented, it can represent a high estimate of what we can expect as the final extent of the real human miRNome.

 For the first time, miRNA was discovered in 1993 and seemed to be involved in *C. elegans* developmental timing (Elegans heterochronic gene lin-4, that encodes small RNAs to lin-14 with antisense complementarity). It has been understood that miRNAs are expressed in every eukaryotic organism and are involved in almost all cellular functions. miRNAs inhibit the translation of their targets or mediate mRNA decay.^31^ miRNA affects mRNAs and could eliminate the 3_ polyA tail as well as 5_cap, which in turn degrades and destabilizes this mRNA.^32^ When miRNAs hinder translation without mRNA decay, they often avoid the initiation stage of translation but can influence translation postinitiation.^33^

 Approximately 60-100 nt pri-miRNAs are transcribed by RNA polymerase II.^34^ Drosha (an RNAse III endonuclease) processes Pri-miRNAs, which are recruited co-transcriptionally.^35^ The Drosha complex is linked to 20 polypeptides, known as the Drosha microprocessor complex.^36^ The Drosha cofactor DiGeorge critical region 8 (DGCR8) is a double-stranded RNA binding domain protein (dsRBD), which is necessary to cleave pri-miRNA into 60-70 nt imperfect hairpin precursor-miRNAs (pre-miRNAs). Drosha can be stabilized by DGCR8 and controls the levels of DGCR8 by cleaving DGCR8 mRNA.^37^

**Figure 1:**
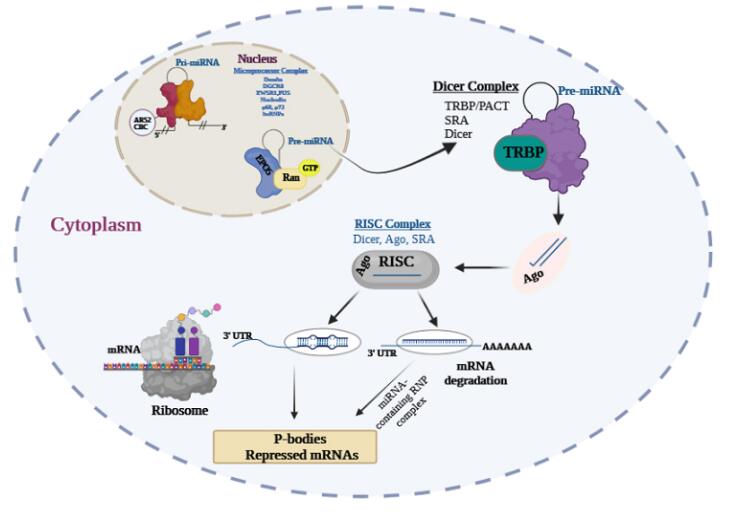
Processing pathway of miRNA: The figure reveals the processing of miRNA. miRNAs are converted to pri-miRNAs by RNA polymerase II, which is processed into precursors – pre-miRNAs by the Drosha microprocessor complex, which contains DGCR8. The p72 and DEAD-box RNA helicases p68 belong to the Drosha complex. Exportin-5 recognizes Ran-GTP and Pre miRNAs with GTP hydrolysis sent to the cytoplasm. Pre-miRNA is processed to mature miRNA by the Dicer complex in the cytoplasm. The TRBP/PACT complex interconnects with Dicer and stabilizes it, then transfers miRNA to Ago proteins in the RISC. miRNA -by binding to the 30 untranslated regions - conductors the RISC complex to target mRNAs and lead to inhibition of the translation. The Silent mRNA degradation occurs in P-bodies

 The DEAD-box RNA helicases p68 and p72 are the components of the Drosha complex that have roles in processing a subset of miRNAs.^38^ Exportin-5 and Ran-GTP export Pre-miRNAs to the cytoplasm. miRNAs are processed by the Dicer complex inside the cytoplasm. Dicer (a cytoplasmic RNAse III) removes the loop structure of pre-miRNA to create the mature ~22 nt miRNA duplex.^39^ Dicer transfers miRNA to Argonaute proteins (Ago1, Ago2, Ago3, and Ago4), TRBP, and PACT in the RNA-induced silencing complex (RISC).^40^ Ago proteins change the miRNA duplexes and form products like single-stranded miRNA-5p and miRNA-3p. Complementary binding of the mature miRNA to the 3-untranslated region of a target mRNA, and its association with the RISC, result in translational mRNA degradation or repression^41^ (Figure 1).

 It has been discovered that more than 2000 miRNAs exist in human cells, most of these miRNAs are specific in some cells, tissues, or organs.^42^ They control many biological procedures, like cell differentiation and proliferation, growth control, apoptosis, and development.^42,43^

 Studies have shown that a wide range of human diseases are related to changes in the expression of miRNAs, and in many cases these disorders are considered as a cause in the progression of diseases. Therefore, extensive efforts have been made to develop miRNA therapy, depending on For the purpose of treatment, these studies have been conducted on inhibitors and enhancers of miRNAs, and the most promising drugs that have been introduced so far are drugs such as: Regulus RG-101, which is an inhibitor of miR-122 and it is used to treat HCV infection,^44^ or drugs such as let-7, which are used to treat cancer diseases, or the use of miR-34 in cancer and cardiovascular diseases, as well as the use of miR-29 for fibrosis.^45,46^

 Mechanisms for these therapeutic goals have been introduced, which fall into two general categories: therapeutic strategies for miRNA-decreasing diseases and therapeutic strategies for miRNA-enhancing diseases, which in the first category substitute a miRNA mimetic for diseases in which miRNAs are decreased. It is found that this imitation is usually a dsRNA duplex that is loaded in RISC or a stem loop precursor is loaded in RISC, but in the second category where the increase of miRNAs occurs in diseases, oligonucleotide inhibitors can be used. which, as an inhibitor, prevents the binding of miRNA to the target miRNAs by entering the RISC complex.^45,47^

 Studies show that miRNAs are involved in regulating brain development and disorder.^48^ However, the practical treatment based on the use of microRNAs for neurological diseases has not yet been introduced, and most of the studies conducted have been aimed at proposing strategies and treatment solutions needed for this issue, so we also tried in this review. By examining the various therapeutic aspects of microRNAs and the role and beneficial effects of estrogen use, let’s give the researchers in this field a good view in choosing a more effective and efficient treatment. (Table 1)

**Table 1:** The most abundant miRNAs in the brain

**miRNA**	**Main findings**	**Cell lines**
miRNA-9	miR-9 appropriate differentiation of Cajal-Retzius cells.^49^ miR-9-2 was shown to be upregulated during *in vitro* neuronal differentiation and downregulated in 50% of primary neuroblastoma tumors.^50^	P19 Mouse cortical cells SK-N-BE cells
miRNA-124	A role for miR-124 in regulating the regeneration of a functional brain and visual system.^51^ Overexpression of miR-124 led to an enhancement of neuronal incorporation and differentiation of neural-specific exons in some genes.^52^ Increased levels of miR-124 in neuronal differentiation induced by retinoic acid.^53^	Planarian *Schmidtea mediterranea* mouse neuroblastoma cell lines human NTera2D1 and rodent P19D and Neuro2a (N2a) cells
miRNA-128	mir-128 can inhibit the proliferation of glioma cells by negatively regulating one of its targets, E2F3a, which is highly expressed in glioma and important for cell cycle progression.^54^ miR-128 up-regulation inhibits DCX and Reelin expression and reduces neuroblastoma cell invasiveness and cell motility in cell lines.^55^	T98G glioma cells SH-SY5Y neuroblastoma cells
miRNA-10	inhibition of miR-10b upregulated in both high-grade and low-grade gliomas reduces glioma cell growth by apoptosis and cell-cycle arrest	Human glioma A172, U87, LN229,U251, and neuroblastoma SHSY5Y
miRNA- 29	miR-29a/b reduces mushroom-shaped dendritic spines on hippocampal neurons with a concomitant increase in filopodial-like outgrowths, suggesting an effect on synapse formation via actin cytoskeleton remodeling.^56^	Hippocampal neurons of neonatal C57BL6 mice
let-7b, let-7d	Upregulated in the hippocampus of sleep-deprived rats.^57^	The hypothalamus, hippocampus, occipital cortex, prefrontal cortex, and somatosensory cortex of male rats
miRNA-29	miR-29 expression was downregulated interleukin-23 (IL-23) directly and upregulated in human dendritic cells (DCs) in response to NOD2 signals and IL-23p19 likely via reduction of ATF2.^58^	CD14 + monocytes of mice
miRNA-132	The overexpression of miR-132 increased synaptic MMP-9 level provokes enlargement of the dendritic spine heads.^59^ miR-132 expression influences neuronal maturation via its effects on dendritic and spinogenesis arborization.^60^	Cultured hippocampal neurons from Fmr1 KO mice CA3, CA1and GCL excitatory cell layers of the C57/Bl6 mice hippocampus
miRNA-134	miR-134 Regulates Ischemia/Reperfusion Injury-Induced Neuronal Cell Death by Regulating CREB Signaling.^61^ miR-134 is a negative posttranscriptional regulator of GluA2 expression in Hippocampal Neurons.^62^	Human embryonic kidney 293 (HEK293) cells Hippocampal and primary cortical neurons from embryonic day 18 (E18) Sprague-Dawley rat
miRNA-219	miR-219 cascade regulates neural stem cell proliferation in schizophrenia model and neurodevelopment.^63^	Neural stem cells of Embryonic mouse
miRNA-425	Mir-425 deficiency promotes dopaminergic and neurodegeneration necroptosis in Parkinson’s disease.^64^	Pheochromocytoma PC12 cells of C57BL/6 mice
miRNA-181	Down-regulated hsa-miR-181a and hsa-miR-181b contribute to the malignant appearance in human gliomas.^65^ miR-181a may be involved in a complex feedback loop with cocaine-responsive plasticity genes.^66^	Human glioma cell lines, U251, U87 and TJ905 HEK-293 cells or Male Wistar rat brain parts
miRNA-15	miR-15a is a target of PPAR- transrepression directly regulates BCL-2 and contributes to PPAR-mediated vascular protection against ischemia-like insults.^67^ miR‐15a regulates oxygen-glucose deprivation/reperfusion (OGD/R)‐induced neuronal injury by targeting BDNF.^68^	Cerebral cortex from adult male C57BL/6J mice Primary cortical neurons of Sprague‐Dawley rats, 16‐18 days of age
miRNA-200	miR-200 family in regulating the proliferation and differentiation of neurons.^62^	PC12 cells of rat brain
miRNA-125	miR-125b Overexpression led to an enhancement of neuronal proliferation and differentiation in neural stem/progenitor cells.^58^ miR-125a Increased neuronal differentiation levels induced by retinoic acid.^57^	Neural tissue samples from the hippocampus of newborn Sprague–Dawley rat Mouse P19 embryonal carcinoma cells
miRNA-106	miR-106b may affect neuronal survival by regulating TGF-β signaling through TβR II.^69^	HEK-293T, SH-SY5Y, and CHO cell lines in a double transgenic mouse model for Alzheimer's disease
miRNA-210	The Notch signaling pathway up-regulation can be activated by miR-210 which contributes to angiogenesis after cerebral ischemia.^70^ miR-210 overexpression induces angiogenesis and neurogenesis.^71^	HUVE-12 cells in adult male Sprague–Dawley rats Left basal ganglia of normal adult mouse brain
miRNA-324	miR-324-5p inhibits the proliferation of glioma by target regulation of glioma-associated oncogene 1.^72^	U87 and LN229 cells
miRNA-138	Inhibition of miR-138 led to an increase in dendritic spine volume.^73^	Hippocampal and primary cortical neurons from embryonic day 18 (E18) Sprague–Dawley rats
miRNA-338	miR-338-3p can restrain cell invasion and migration and inhibit cell proliferation by inducing cell cycle arrest, as well as affect the PTEN/Akt pathway by down-regulating PREX2a gene in neuroblastoma tissue sections from patients with metastasis.^74^	Human SH‐SY5Y neuroblastoma cells
miRNA-339	miR-339-5p inhibits alcohol-induced brain inflammation by regulating the NF-κB pathway.^75^	Primary microglial cell cultures of C57BL/6 mice

 Initial studies show that miRNAs are expressed and enriched in region-specific areas of the brain.^76^ miRNAs have important roles in different neurologic processes including neuronal development (miRNA-10, miRNA-9, miRNA-430),^77^ and neuronal cell maintenance (miRNA-29, miRNA-134).^56,78^ The most abundant miRNAs related to neurons include miRNA-124, miRNA-9, miRNA-128, miRNA-29, let-7, and miRNA-26.^79,80,81^

 A miRNA that is especially expressed in the central nervous system is miRNA-124,^82^ and is predominantly expressed in post-mitotic neurons.^83^ During brain development, the expression of miR-124 increases.^84^ miRNA-124 regulates neuronal differentiation and maturation by the expression of small GTPase Ras homolog growth-related (RhoG),^85^ LIM/homeobox protein 2 (Lhx2),^86^ Rho-associated coiled-coil-containing protein kinase 1 (ROCK1),^87^ and cAMP response element-binding (CREB) protein.^88^ It has been shown recently that miR-124 regulates cognition and synaptic transmission by restricting the expression of early growth response gene 1 (Egr1).^88^

 Another miRNA during brain development is miR-132, which regulates neuronal structure^78^ and can contribute to spine morphogenesis,^89^ arborization, and dendritic growth in response to neuronal activity.^90^ miR-132 exerts its anti-inflammatory effect by targeting acetylcholinesterase.^91^ miRNA dysfunction leads to neurodegenerative diseases and acute neurological injury.^92^ In hippocampal neurons, the downregulation of miRNA-132 results in decreased dendritic arborization, while miRNA-132 overexpression increases the likelihood of synaptic transmission.^93^ These studies show that miRNA-132 is one of the essential regulators of synaptic plasticity.

 Neural differentiation is also triggered by miRNA-9 from embryonic stem cells.^94^ miRNA-9 has a pro-differentiation role, i.e., miRNA-9 targets forkhead box protein G1 (FoxG1) expression by Cajal–Retzius neuronal differentiation.^49^ Moreover, miRNA-9 increases the axonal projection and differentiation of spinal motoneurons by targeting forkhead box protein P1 (FoxP1).^95^ miRNA-9 also restricts the expression of forkhead box protein P2 (FoxP2) and triggers the maturation and migration of cortical neurons.^96^ miRNA-9/9 in cooperation with miRNA-124 can amplify the crucial role of miRNA-9 in neuronal differentiation by transforming human fibroblast cells into functional neurons.^97^

 An important miRNA in neuronal plasticity is miRNA-134 which is shown to be localized to the synapse in the hippocampal neurons of rats. miRNA-134 is regulated by the protein deacetylase SIRT1. Knockdown of SIRT1 results in elevating miRNA-134 expression as well as decreasing CREB and synaptic plasticity.^98^

 One of the brain miRNAs that play a pro-differentiation role is miRNA-137 which is found in the neural stem cells of embryonic^99^ and adult mouse brains.^100^ High miR-137 expressions increase neural differentiation and reduce neural stem cell proliferation. A miRNA that is specifically expressed in mammalian brains is miRNA-219. This miRNA is essential and adequate for increasing the differentiation of oligodendrocytes in the mouse by inhibiting negative regulators of oligodendrocyte differentiation.^101^

 Studies have demonstrated that miRNAs are an important reason for neurodevelopmental, neuropsychiatric, and neurodegenerative diseases.^102^ Evidence suggests that a change in miRNA-137 expression levels influences functional connectivity and brain activation in individuals with the risk of schizophrenia.^103^ The pathological importance of miRNA-124 and miRNA-9 and their target genes in schizophrenia disorder deserves further investigation.^104^ Studies show that the expression of miRNA-132 is considerably reduced in the brains of patients with schizophrenia.^105^

 In the study on the patients with schizophrenia, bipolar disorder, and psychiatrically normal control subjects, compared to the control group in the schizophrenia patient group, all miRNAs reduced expression and exhibited lower expression in the bipolar disorder patient group. These miRNAs include miRNA-425, miRNA-33, miRNA-106b, miRNA-138, miRNA-22, miRNA-151, miRNA-210, miRNA-324-5p, miRNA-338, miRNA-339, and miRNA-15a.^106^

 Profiles of miRNA expression in the brains of old and young mice revealed three miRNAs called miRNA-34, miRNA-124, and let-7, which were changed as a function of age.^107^ Evidence of increased miR-34a expression in the brain, as well as the blood of older mice, could be a potential marker of brain aging in mice, implying that miRNAs can serve as a suitable biomarker of brain aging in humans.^108^ Specifically, the study showed that miRNA-124, miRNA-181a, miRNA-9, miRNA-125a, and miRNA-29 changed with aging in the brain.^67^

## Human organs and estrogen synthesis, distribution, and receptors

 Estrogen synthesis, in addition to the ovaries, is seen in different parts of the brain, adipose tissue, Vascular endothelium, and muscular cells as well as osteoblasts.^109^

 Which produces estrogen in these tissues depends on the availability of these tissues to C19 steroid precursors.

 also, the amount of estrogen metabolism in extra gonadal tissues can be affected by different distributions of enzymes. with age, the secretion and plasma concentration of estrogen decreases significantly.

**Figure 2:**
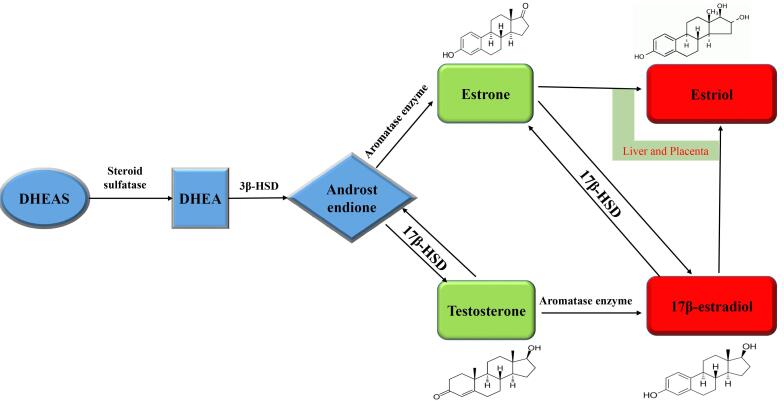
Schematic of estrogen synthesis. DHEAS – dehydroepiandrosterone sulfate; DHEA – dehydroepiandrosterone; 3β-HSD – 3β-hydroxysteroid dehydrogenase; 17β-HSD – 17β- hydroxysteroid dehydrogenase

 The presence of large amounts of DHEA and DHEAS in the circulation indicates the presence of a large reservoir of estrogen and testosterone precursors in extra genital tissues (Figure 2).

 Estrogens exert all their effects through their three known receptors. The type of alpha (ERα) before the other receptors was discovered and investigated and with a significant interval after that, the type of beta (ERβ) was discovered with distinct roles.^110^ The third type of receptor is a G protein-coupled estrogen receptor 1 (GPER1). ERβ at least five and ERα has at least three different isoforms. ERα36 isoform is able to interact with GPER1.

 ERβ isoforms due to the inability of transcriptional activity, dimerize with ERα and silencing ERα signaling. Estrogen signaling can be greatly affected by these isoform differences.^74,111^ The response to different ligands depends on the concentration of the ERα and ERβ and their expression is different in various organs and tissues.^112^ GPER1 is a plasma membrane receptor that is responsible for estrogen signaling quickly and is expressed independently of other receptors.^111^ has a high affinity for estrogen, especially 17β-estradiol and activates adenylate cyclase, and finally increasing levels of cAMP.^113-115^ other steroid hormones have very little tendency to bind to the GPER1.^116,117^

## Comprehensive role of estrogen in health and neuroprotection

 Three major endogenous estrogens in females include 17β-estradiol (E2), estrone (E1), and estriol. E2 is the predominant estrogen in humans, the main estrogen in premenopausal women (menopausal transition) that is synthesized from cholesterol in the ovary in response to luteinizing hormone (LH) secretion. In postmenopausal women, E1 is the major estrogen, which is synthesized from adrenal androgens in adipose tissue. Furthermore, E1 and E2 can be synthesized in special tissues, for example, in the breast^118^ and lung.^119^ These hormones regulate development, fertility, homeostasis, survival, and proliferation in multiple tissues like the cardiovascular system, brain, skin, breast, bone, intense, lung, and reproductive tract in both men and women.^120^

 Estrogens originate from cholesterol and, in the mitochondria, are converted into 17α-hydroxypregnenolone. In the next step, they are transferred to the smooth endoplasmic reticulum to form dehydroepiandrosterone and, after that, to androgens like testosterone and androstenedione.^121^ Postmenopausal women seem to be at higher risk for some diseases like heart disease, dementia, and osteoporosis because of a lower level of estrogen.^122^ Shreds of evidence suggest that all the enzymes required for E2 production are available in the brain and help region-specific estrogen production.^123^

 With the involvement of the aromatase (ARO) enzyme, estrogens are produced from androgens in the CNS of men and women.^124^ For the final conversion of testosterone into E2 and androstenedione into estrone, a key enzyme is involved which is cytochrome P450. Studies have shown that ARO affects the GnRH/LH surge and produces local estrogen in females; it can also affect neuronal plasticity in the amygdala and hippocampus.^125^

 Estrogen receptors do their function through two main pathways: classical (genomic) or non-classical (non-genomic or rapid signaling). Estrogen in the classical signaling pathway initiates transcription in the target gene by binding to an estrogen receptor (ER), normally ERα or ERβ, which translocates the nucleus and binds to an estrogen response element (ERE). Both ERα and ERβ are extensively expressed normally in all cell types overall the brain, for example, endothelial cells, glia, neurons, and areas that are sensitive to ischemia like the hippocampus and neocortex.^126^

 Moreover, non-classical estrogen signaling is through G-protein coupled estrogen receptor 1 (GPER1), which is located on intracellular and plasma membranes and has been identified to mediate neuroprotective and anti-apoptotic effects in the hippocampal and cortical neurons of mice.^127,128^

 Recent studies have represented that estrogen provides benefits that positively influence acute injury and plasticity as well as regeneration of neurons after ischemia.^129^ It can improve memory function in animals receiving estrogen supplementation after ischemia.^130^ Estrogen triggers a cascade of subcellular actions after an ischemic injury, which includes both genomic and non-genomic mechanisms like stabilizing the blood-brain barrier,^131^ reducing the brain edema,^132^ vasculature dilating, increasing cerebral blood flow, inflammation suppressing,^133^ and upregulating cell survival mediators.^134^ Moreover, estrogen has an antioxidant effect and prevents lipid peroxidation.^135^

 Activating the receptor of N-methyl-D-aspartate may be involved in neuroprotection via estrogen. Estrogen,^136^ like estrone and estradiol, may be able to improve antioxidant effects in human neuroblastoma cells (SHSY5Y) and neuronal cultures via increasing ATP levels, mitochondrial membrane potential (MMP), and manganese superoxide dismutase (MnSOD) activity,^137^ which is a very important modulator of the brain metabolic system and may contribute to processes in all the bioenergetic system including metabolism and glucose transport, as well as mitochondrial respiration and ATP production.^138^ Nowadays, it is recognized that the nuclear transcription of different proteins can be regulated by estrogen which affects the function of mitochondria such as nuclear respiratory factor-1 and peroxisome proliferator-activated receptor-gamma coactivator-1.^139^ Therefore, this function is crucial for activating genes encoding proteins, that are involved in the mitochondrial electron transport chain complexes and mitochondrial biogenesis.

 Menopause is a period of women’s life that influences their physiological and psychological conditions and brings some problems for their quality of life.^140^ The levels of sex hormone-like estrogen and progesterone decrease in this part of their life. Menopause has some symptoms, one of which is memory problems and cognitive impairment.^141^

 It is recognized that during menopause, the antioxidant enzyme systems (e.g., superoxide dismutase and glutathione peroxidase), and antioxidant vitamins (e.g., vitamin C and vitamin E) are reduced, which is associated with the high level of oxidative stress.^142^ In the nervous system tissue, estrogen limits cell death by decreasing elevated intracellular Ca^2+^. This element has an important role in extending ischemic damage induced by ROS. Antioxidant effects of estrogen hinder neurodegeneration, particularly in the hippocampus through different mechanisms,^143^ This could clarify how estrogen improves cognitive functions.^144^

 The formation of new synaptic connections is another effect of estrogen on the CNS.^145^ OVX due to estrogen deficiency affects learning and memory.^146^ Estrogen can increase the amount of brain-derived neurotrophic factor (BDNF) in the CNS, specifically the hippocampus.^147^ It can be effective in the formation of synapses by altering ERαs expression.^148-150^ because it has been demonstrated that ERs regulate synaptic protein levels in CA3 and CA1 areas in the hippocampus of humans.^151^

 Estrogen activates some anti-inflammatory factors like GPER1. GPER1 inhibits nuclear factor kappa beta (NF-κB) and its target genes (e.g., TNFα and IL-1β) that lead to neuroprotection in rats with cerebral ischemia.^152^ In addition, activated GPER1 via estrogen can improve the function of tight junctions, and reduce the permeability of the blood-brain barrier.^153^ The timing and dose of estrogen therapy are really important for achieving the required neuroprotective effects.^154^ Neuroinflammation parameters that increase in ovariectomized mice hippocampus, like microglia activation and NF-κB, are also estimated for discovering mood disorders.^155^

 Furthermore, the anti-inflammatory effect of estrogen is essential for CNS development (proliferation or differentiation of neural stem/progenitor cells)^156^ and important for synaptic plasticity regulation.^157^

 Researchers have also recognized the effects of estrogen on modulating mood in females.^158^ Mood disorders increase during menopause in women because of estrogen reduction and two predominant symptoms are depression and anxiety.^159^ The low estrogen levels at the diestrus stage lead to depression-like behaviors in rodents,^160^ estrogen deficiency can also cause depressive behaviors in ovariectomized rats.^161^

## Estrogen’s multifaceted impact on miRNA regulation and brain function

 Even though the number of investigations in miRNAs in animals and humans has increased rapidly in these years, there are still a few studies on miRNA regulation by estrogen. Some steroid hormones, like testosterone, progesterone, and estradiol, regulate miRNA expression.^162^ As we know, miRNAs have their own promoter elements or are placed on their host genes. It is proven that E2 signaling changes the expression of Dicer, Drosha, and Argonaute and, thus, can regulate the maturation of miRNA.^163^ Furthermore, sex hormones regulate miRNAs directly via binding to the promoter of miRNA elements or indirectly via binding to nuclear hormone receptors. Consequently, this ligand binding to the nuclear receptor regulates multiple gene transcription and/or recruits coactivators or corepressors.^164^

 There is a different sex-specific model of miRNA expression in the brains of rodents. Blocking the conversion of testosterone into estradiol in men causes this pattern to disappear, showing that miRNAs are regulated by estrogens.^165^ miRNA-23a expression is also dependent on sex differences and its effect can be related to the sex-specific activation of cell death in a cerebral ischemia model.^166^ Furthermore, miRNA-101a and miRNA-199a are shown to be the regulators of COX-2 in the developing preoptic area, which is induced by E2 and is required to differentiate the density of the dendritic spine in this region in different sexes.^167^

 Taken together, all these studies demonstrate that tissue-specific miRNAs can be regulated in a differential procedure throughout the development process in the brains of males and females. Consequently, the changes in gene and protein expression can alter the outcomes of some diseases that are associated with sex differences, like stroke. Experimental studies show that some members of the miRNA-200 family such as miRNA200b-3P, miRNA-141-3p, miRNA-429, and miRNA200a-3P have a sex-specific expression pattern in cortex regions of the brain. This study showed that male rats had lower levels of these miRNAs than females in the P0 area, but in the P7 area, females had lower levels of miRNAs than males and adults.^168^

 MiRNAs regulate cell processes that are important for brain sexual differentiation. For instance, the anteroventral periventricular nucleus in the brain has a bigger size in females than in males.^169^ It means that E2 activates the apoptotic pathways via miRNA regulation.^170^ Most of the miRNAs are involved in regulating the apoptotic process.^171^

 The gonadal steroid hormone E2 plays an important role in regulating biochemical processing and miRNA expression in some systems, such as models of cancer cells.^172^ Sufficient evidence has shown that miRNAs respond to estrogen. A study on zebrafish showed that microRNA can be regulated by estrogen.^173^ which is cell type-specific, e.g. miRNA-196b that targets Hoxb8a directly and is increased with E2 in the skin but is down-regulated in the liver and intestines.^174^ Moreover, E2 can regulate miRNA expression differently in abnormal and normal tissue; for example, E2 downregulates the expression of miRNA-26 in leiomyoma cells but increases this miRNA in myometrial cells.^175^

 The let-7 miRNA family is up-regulated by estradiol.^176^ Estrogen reduces proliferation and increases neuronal differentiation in neuroblastoma via let-7.^177^

 Research has shown that E2 has a neuroprotective effect by reducing the time of recovery after the stroke and improving memory and learning in aged humans, rodents, and primates.^178^

 Increasing age causes worldwide changes in the expression of the neuronal gene. Recent investigations have shown that the expression of miRNA changes with age in the aging female brain as a result of decreasing estrogen. Age-related miRNA changes can be responsible for regulating all the changes in gene expression.^107^

 One of the findings from studies has been that E2 can control hippocampal-mediated emotion or stress via differentially regulating miRNA-9 and miRNA-9-3p. They have reported that E2 decreases miRNA-9 expression in the dorsal hippocampus of 3-month-old rats, but increases at the same time miRNA-9-3p expression in animals.^179^ They have also shown that a subset of miRNAs in the ventral hippocampus like let-7i, miRNA- 495, miRNA-125a, miRNA-9-3p, miRNA-9, miRNA-181a, and miRNA-7a is regulated by E2 in an age-dependent pattern.^179^

 In other studies, 18-month-old female rats are ovariectomized and, then, an acute estradiol treatment is prescribed at 1, 4, 8, or 12 weeks after OVX. Also, the expression of several miRNA processing proteins is measured. Taken together, their results- in the aged hypothalamus- reveal that long ovarian hormone deprivation changes the E2 regulation of mature miRNAs. Analysis of this study suggests that E2 raises the expression of mature miRNA-9, miRNA-7a, let-7i, miRNA-181a, and miRNA-9-3p one week after OVX. However E2 treatment decreases miRNA-495 levels at 12 weeks after OVX. Analyses have demonstrated that six out of seven miRNAs such as let-7i, miRNA-495, miRNA-125a, miRNA-181a, miRNA-9-3p, and miRNA-9 are increased as the result of age alone. Specifically, other results show that expression of let-7i increases at 12 weeks after OVX in vehicle-treated animals, and miRNA-181a, miRNA-9-3p, miRNA-125a, miRNA-495, and miRNA-9 increase in both vehicles- and E2-treated animals at 8 and 12 weeks after OVX. Recent studies have demonstrated that this miRNA plays a substantial role in brain and nervous system development^180^ and has tumor-suppressor-like properties in glioblastomas.^181^

## Overall insights into estrogen signaling and epigenetic regulation

 We have two types of estrogen-dependent signaling. Genomic and non-genomic and the estrogen-ER complex to DNA can be done both directly and indirectly. The direct pathway is the usual signaling path that estrogen-ER complex acts as a transcriptional activator promoting gene expression.^182^ In the indirect type of signaling path, ligand-activated ERs bind to DNA through protein-protein interactions with other classes of transcription factors.^183^

 Non-genomic effects of E2 usually occur within seconds.^184^ This fast activity is created by an ‘orphan’ GPR30 or plasma membrane (PM)-associated ER.^185^ Molecular mechanisms of the non-genomic signaling depend on the variety of cells, numerous, diverse, and activated with various protein-kinase cascades. It is also associated with the type of cellular receptor (ERα, ERβ, and GPER1). ERs can also be activated via the general Phosphorylation state of the receptors such as protein kinase C or protein kinase A, in the absence of 17β-estradiol or another suitable ligand. this way is called the Ligand-independent signaling method. Studies have shown that conjugates with E2 initiate intracellular kinase cascade activities including PI3K/AKT and MAPK/ERK rapidly.^186-188^

 in recent years, another group of important regulators of gene expression has received special attention as epigenetic mechanisms, including the methylation of miRNA, DNA, and histone modifications. Most posttranslational modifications of histone proteins including phosphorylation, deamination, acetylation, ubiquitination, and methylation are dynamic.^189,190^ Estrogen signaling in addition to gene activation. In addition to coactivators, corepressors are also very effective in suppressing and activating genes involved in the estrogen signaling pathway.^74,110^ Generally, chromatin modifications are necessary for estrogen-mediated transcriptional gene expression. Epigenetic mechanisms have an important role in the regulation of estrogen signaling and the control of ER gene expression. Similarly, miRNAs such as miR-22, miR-18a, miR-221/222, and miR-206 were also implicated in ER gene expression in normal and tumorous cells.^191,192^ Estrogen signaling is also able to regulate the expression of miRNAs and certain chromatin-modifying enzymes.^174^ Finally, miRNAs identified as targeting GPER, and ER are respectively, miR-424 and miR-92.^193,194^

 Studies have shown that exposure to different doses of estrogen causes hypomethylation or hyper-methylation of genes, resulting in suppression or activation of certain genes.^195-198^ Generally epigenetic regulation and Estrogen signaling are two regulatory mechanisms essential for tissue homeostasis.

## Estrogenic regulation of miRNAs for neuroprotection in major brain disorders

 Estrogen has neuroprotective effects on various major diseases and conditions, like Alzheimer’s disease (AD),^199^ Parkinson’s disease,^200,201,202^ multiple sclerosis (MS),^203^ spinal cord injury,^204^ ischemic stroke.^205^ and retinal degeneration.^206^ The human lifespan is increasing with each generation due to an increase in age-related disorders. Studies have shown that E2 of less than normal levels is one of the reasons that cause women after menopause to have risks of acquiring mild cognitive impairments, dementia, and AD. ^206^

 Studies have suggested that estrogen has neuroprotective roles against AD-related pathology; these beneficial effects are directly connected to decreased amyloid-β (Aβ) peptides and pathologic aggregates of tau protein,^207^ which is one of the properties of this hormone. Estrogen therapy decreases Aβ-induced neuronal death via ERα-dependent signaling pathways.^208^ Researchers have also recognized that estrogen receptors can regulate the expression of miRNAs that are involved in modulating the phosphorylation state of tau. ERα and ERβ have reverse effects on regulating miRNA-218/ protein tyrosine phosphatase α (PTPα) signaling. miRNA-218 targets PTPα and reduces its levels with increasing tau phosphorylation. ERα and ERβ are shown to increase and attenuate miRNA-218 levels in HEK293/tau cells overexpressing ERα or ERβ, respectively.^209^ miRNA-218 can be expressed in the hippocampus^210^ and is activated during neuronal differentiation.^211^

 Another miRNA that is involved in AD is miRNA-106a/b which is expressed abundantly in the hippocampus.^68^ It is recognized that there is an opposite relationship between the miRNA-106a expression and STAT3 in the hippocampus of ovariectomized mice. Moreover, miRNA-106a is able to inhibit directly the expression of STAT3. Hence, estrogen can indirectly upregulate miRNA-106a in mice 12 weeks after OVX. miRNA-106 regulates negatively cholesterol efflux via the target gene ATP-binding cassette TransporterA1(ABCA1) as a therapeutic target for AD^212^ and may help regulate β-amyloid precursor protein (APP) expression in the brain and differentiate the neurons.^213^ Increasing APP protein levels cause high levels of Aβ and, consequently, synaptic dysfunction, neurodegeneration, and cognitive impairment.^214^

 MS is one of the CNS inflammatory autoimmune disorders, a classical autoimmune disease that results in injury to oligodendrocytes, loss of myelin, and neurological dysfunction.^215^ Studies have clarified selective estrogen-receptor modulators increase CNS remyelination independent of estrogen receptors.^216^ Previous studies have revealed six miRNAs (miR-422a, miR-572, miR-1826, miR-614, miR-648, miR-1826, miR-22, and miR-614) that are implicated in MS.^217^ Among these miRNAs, miR-22 may inhibit estrogen signaling by targeting the estrogen receptor alpha mRNA. However, the estrogen-MS signaling remains unclear.^218^

 CNS injuries like traumatic brain injury, acute ischemic stroke, and SCI increase the risk of death and disability throughout the world.^219^ Studies further elucidate that progesterone and estrogen play a key role in neuroprotection after ischemia in ovariectomized female animals.^220^ Furthermore, in male rats with diabetes, estrogen reduces ischemic stroke infarct size.^221^ miRNA dysregulation may contribute to the outcome after stroke.^222^ Estrogen also suppresses NF-κB activation via controlling miRNA-125b and let-7a that regulate expression KappaB-Ras2 levels.^223^ Recent studies show that inhibiting miRNA181–5p has protective effects on focal ischemia in the female mouse with focal ischemia, which can be via balancing estrogen receptors present on astrocytes.^224^ Whilst previous studies have identified several miRNAs, many studies still need to explain the role of estrogen in the regulation of miRNAs in ischemic stroke or other diseases in the brain.

 Studies show that in general, the expression of both types of estrogen receptors (ERα and ERβ) increases during neural development,^225,226^ but after birth, the expression of these receptors decreases in the brain and with a wide distribution pattern. It is limited to certain areas of the brain.^227,228^ With the onset of old age, systemic and specific changes occur in the brain and the flexibility of the brain against side effects is affected, and changes in the level of estrogen receptors are not excluded from this issue, and it has been shown that there is a significant decrease with age. It is seen in the levels of ERα and ERβ in the synapses of CA_1_ neurons in the hippocampus of female rats.^227,229^ Therefore, we will lose a level of neuroprotection during this period. Menopause, we see that it seems that this makes the risk due to the lack of this defense barrier double in old women. For example, memory problems and mood changes after menopause in women, as well as the higher prevalence of Alzheimer's disease in women than in men, are related to estrogen deficiency. Due to the inflammatory nature of Alzheimer's disease, it seems that estrogen levels and increasing the duration of estrogen use may affect the process of Alzheimer's disease through neurotrophic, anti-inflammatory effects, increasing cerebral blood flow and reducing the formation of f3-amyloid and increasing the expression of apolipoprotein E gene. Make it more resistant to the disease process.^230-232^ Estrogen has also been shown to delay or prevent the onset of Parkinson's disease, and several mechanisms have been proposed in animal models for this, including: the positive and protective effect of estrogen on dopamine neurotransmission as well as the reduction of dopamine reuptake in synapses. Reducing the response threshold of L-DOPA and increasing the sensitivity of neurons to dopamine.^233,234^ Therefore, it seems that by understanding the spatial and temporal expression and signaling of estrogen receptors, we may gain a new perspective on the pathological and physiological aging of the brain, and the gender differences in this process will also be revealed.

 Since brain aging and various brain diseases such as Alzheimer’s etc are thought to result from an imbalance between nerve repair and damage, estrogen seems to play a significant role in slowing down these processes and repair, dendritic branching, and survival. have neurons Past studies have stated various mechanisms for exerting these estrogen effects, including: through a direct effect on estrogen receptors located in neurons of different brain regions and effects such as: increasing the concentration, release, reabsorption and inactivation of neurotransmitters such as dopamine, serotonin and norepinephrine in nerve synapses, increasing the number of receptors of these transmitters,^220,232,235,236^ and also through binding to endothelium receptors and stimulating the release of nitric oxide, which causes vasodilation and increases blood supply to the brain (which is the brain for metabolism It is very dependent on this blood flow) and prevents plaque formation and finally leads to its own protective and neurotrophic effects in the brain.^233,237-239^

## Conclusion

 Recent studies show that miRNAs have emerged as novel regulatory mechanisms in pathological and physiological processes by modulating gene expression profiles at the post-transcriptional level, and miRNAs have also been shown to be potential tools for prognosis or diagnosis. and biomarkers are non-invasive and cost-effective and have provided insights into brain function. However, knowledge about the regulatory effects of estrogen on miRNA profiles in brain function is still scarce. Therefore, new therapeutic approaches attempt to identify new miRNAs. In the pathogenesis of diseases, they have been prescribed or manipulated, among which the importance of estrogen in brain function can also be considered in miRNA-based treatments.

## Competing Interests

 The authors declare no potential competing interest.

## Ethical Approval

 Not applicable.
